# Optimal public-transport operational strategies to reduce cost and vehicle’s emission

**DOI:** 10.1371/journal.pone.0201138

**Published:** 2018-08-01

**Authors:** Chunyan Tang, Avishai Ceder, Ying-En Ge

**Affiliations:** 1 College of Transportation Engineering, Dalian Maritime University, Dalian, Liaoning Province, China; 2 Transportation Research Centre, Department of Civil and Environmental Engineering, University of Auckland, Auckland, New Zealand; 3 Faculty of Civil and Environmental Engineering, Technion—Israel Institute of Technology, Haifa, Israel; 4 College of Transport & Communications, Shanghai Maritime University, Shanghai, China; Chongqing University, CHINA

## Abstract

Public transport passenger demand is inevitably made non-uniform because of spatial and temporal land use planning. This non-uniformity warrants the use of public transport operational strategies to attain operating efficiency. The optimization of these strategies is commonly being done from the operator perspective, and indirectly from the user perspective. However, the environmental perspective of these strategies, in terms of vehicle’s emission, has not been investigated. This study proposed a methodology to analyze the benefits of using transit operational strategies to reduce operating cost and eventually also to reduce undesirable emissions. First, a strategy-based optimization model is established to minimize the number of transit vehicles required. Four candidate operational strategies are considered in this model, including full route operation (FRO), short turn, limited stop, and a combination of limited stop and short turn. Second, the pollutant emissions of transit vehicles are estimated by the MOVES emission model. The developed methodology is applied to a real life case study in Dalian, China. Results show that the use of operational strategies can not only significantly save the number of vehicles by 12.5%, but also reduce emissions of pollutants (i.e., CO_2_, HC, CO, NO_x_, PM_2.5_) by approximately 13%, compared with applying FRO strategy exclusively. In addition, both benefits can be further enhanced through the use of an efficient payment mode (e.g., off-board or contactless card) or improving bus performance in deceleration/acceleration as well as doors opening and closing at a stop.

## Introduction

The rapid growth of private vehicle ownership and usage has been made significant accessibility and convenience for human travel activities. However it also produces a considerable amount of emissions including carbon dioxide (CO_2_), nitrogen oxides (NO_x_), hydrocarbons (HC), carbon monoxide (CO), particulate matter (PM), etc. The International Energy Agency (IEA) estimated transport activity contributing approximately 23% of CO_2_ [1], 11% of PM_2.5_, and 28% of NO_x_. These emissions need to be drastically reduced because of adverse impacts in terms of human health and environment. Currently, the emission-reduction challenge of the transport sector is in an irreversible shift to low-emission mobility. With this in mind, public transport is a sustainable alternative enabling to respond to the increasing mobility needs with lower emissions [2, 3]. Nonetheless, public transport modes of travel have a great potential for further improvement in terms of their operational efficiency as well as environment impacts.

Due to the fact that a common full route operation (FRO) strategy is used for meeting imbalanced travel demand in most cities throughout the world, full advantage is not always taken of public transit resources. This inefficient operation situation will create the undesirable in-vehicle crowding in some segments with high travel demand, excessive empty seats in other segments with low travel demand, and increases of operating cost as well as round trip time. This may not only frustrate passengers, but may also result in a loss of resources and environmental deterioration.

The mismatch between supply and demand becomes more and more apparent in the bus route that partially overlaps with a subway line in urban areas. Because of having the subway with shorter in-vehicle times and higher reliability, passengers tend to use it if their origins, destinations, or both are serviced by the subway. As a result, on bus-route segments not overlapping the subway, passengers’ overcrowding is often observed, whereas on overlapped segments low passenger loads with empty-seats are often observed. In this case, some approaches should be explored to achieve the better match between supply and demand on the bus route. One efficient method in previous studies is to use multiple operational strategies to suit the observed travel demand in the best possible manner, such as limited stop, short turn, deadheading, and mixed strategy. Furth and Day [4] showed the use of operational strategies was able to increase bus productivity significantly with even loads on vehicles that would lead to substantial operating cost reductions.

To a bus route with a high concentration on its some segments, a short turn strategy can be used to reduce the number of vehicles required for covering demand [5–8]. However with regards to routes exhibiting low concentrated but imbalanced, high demand, using a limited stop strategy demonstrates greater benefits than a short turn strategy. Because it is understood that a limited stop strategy servicing a subset of stops along the route produces considerable travel time savings, high average operating speeds [9], and great customer satisfaction [10]. Using archived vehicle location and passenger count data, El-Geneidy and Surprenant-Legault [11] evaluated the application of a limited stop strategy in Montréal, Canada, finding that it yielded 4.6 minutes in running time. While the demand pattern displays imbalances between directions in a bus route, or among routes in a public transport network, implementing a deadheading strategy indicates more benefits for users and operators [12,13].There are cases, however, in which the demand pattern indeed presents imbalances but does not suggest a priori the type of strategy to follow in order to improve transit operation efficiency. In some route segments a short turn strategy could be advantageous, while in the next segment either limited stop or deadheading may be better. In these cases a mixed strategy is worth exploring [14].

Apparently, it has been proven to be more beneficial for a transit operation system when an additional strategy is introduced, not only using FRO strategy. But in practice, most routes present more mixed and complex load patterns. For such demand situations, the implementation of multiple operational strategies appears to a more promising alternative than introducing a single strategy [15–18].

Studies on environment impacts, related to using public transport, focus mainly on the effects of alternative technology and of operation measures. Alternative technology includes bus fuels e.g., diesel, gasoline, biodiesel, compressed natural gas, hybrid[19,20], and electric, bus performance [21], etc. Compared with a diesel bus, an electric bus can reduce 19–35% in CO_2_ emissions through a life-cycle assessment [22]. Electric buses will further benefit the environment if a cleaner power grid and a higher system charging efficiency will be provided in the future. The operation-based improvements consist of transit signal priority, bus stop location, queue jumper lane, types of buses selection, and driver braking [23,24]. During traffic congestion the use of transit signal priority can reduce greenhouse gas emissions by 14%. When considering the combined use of both alternative technology and operational improvements, emission reductions can been further improved to a level of 23% in greenhouse gas emissions [25].

Extensive research has been conducted to investigate the use of operational strategies in improving bus operating efficiency and service levels, but with little attention so far to the emissions performance of these operational strategies, except that Alam and Hatzopoulou [26] applied a linear regressions analysis method to capture the environmental impacts of using a limited stop strategy. There is a need, therefore, to estimate the impacts of using operational strategies on emissions.

Currently, MOtor Vehicle Emission Simulator (MOVES) model is available for the use in transportation projects of actual estimation of environmental impacts[27]. It is a new-generation regulatory emission model developed by the U.S. Environmental Protection Agency (EPA), to replace MOBILE. Compared with MOBILE, the MOVES doesn’t only adopt a modal-based approach to estimate emissions rather than using speed correction factors, but also works with the MYSQL database applied to store input data for supporting emissions estimation and output data for summarizing emissions relevant information. It allows users to freely access this database and revise the input data according to local practical condition. Vallamsundar and Lin[28] used a case study of Cook County in Illinois, U.S., to compare emission estimates of CO_2_ and NO_x_ in both MOVES and MOBILE, finding that the latter had a lower estimate in both pollutants than the former because of the underlying base emission rates. Wallace et al. [29] reported that the observed CO-to-NO_x_ ratio in the winter was more similar to the hourly running emissions ratio predicted by MOVES than MOBILE. In addition, MOVES also was integrated with VISSIM to explore the impacts of primary parameters such as speed, volume, road grade and fuel type, on CO_2_ emissions [30] and greenhouse gas emissions [26].

The main contributions of this work are two-fold: (i) develop an integrated methodology, including an optimization model, to accurately assess the impacts of the use of operational strategies on operating cost and emissions; and (ii) use of strategy-based activated intermediate stops as variables in the optimization model. This study, for the first time, to the best of our knowledge, provides an assessment procedure of emissions of the use of multiple bus-operational strategies. The results will help making appropriate transit polices to handle environmental issues.

The remainder of this study is organized as follows: The methods used for achieving optimal bus operational strategies and for estimating their associated emissions, are presented in Section 2. Then a real life case study from Dalian in China is introduced in Section 3. The results are illustrated in Section 4. Finally, the main findings are summarized, and extensions of this approach are proposed in Section 5.

## Methodology

**Fig 1:**
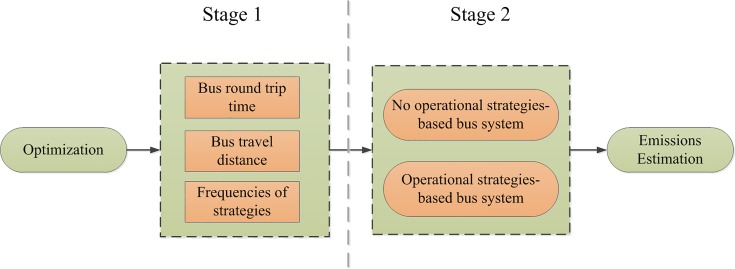
Overview of the methodology; Stage 1: strategies-based operations and Stage 2: emissions estimation.

We propose a methodology consisting of two stages shown in Fig 1 with the purpose to investigate and analyze the benefits of using operational strategies in reducing both the operating cost and the undesirable environmental impacts. That is, through the minimization of the operational cost to measure the reduction attained of the emissions. The first stage is to construct an operational strategies-based bus operations system to obtain the optimal combination of multiple operational strategies. Four feasible operational strategies are included: full route operation (FRO), short turn (ST), limited stop (LS), and a mix of limited stop and short turn (MLS). The second stage uses an emission model to estimate vehicle emissions before and after the implementation of the optimal operational strategies attained.

### Notation

*AT*^*h*^_*l*,*p*_ passenger alighting times of strategy *l* at stop *p* during period *h*

*BT*^*h*^_*l*,*p*_ passenger boarding times of strategy *l* at stop *p* during period *h*

*CT*^*h*^_*l*_ round trip time of strategy *l* in period *h*

*DW*^*h*^_*l*,*p*_ passenger boarding /alighting times of strategy *l* at stop *p* during period *h*

*DT*^*h*^_*l*_ total dwell time of strategy *l* along the bus line in period *h*

*E* set of end stops of strategies

$E_{m}^{total}$ total emissions of pollutant *m*

*EF*_*l*,*m*_ emission factor of pollutant *m* of strategy *l*

*f*_*l*_ frequency of strategy *l*

*H* set of operation periods of a bus line

*L* set of strategies consisting of full route operation (*FRO*), limited stop (*LS*), short turn (*ST*), a mix of limited stop and short turn (*MLS*)

*LH*_*l*_ travel distance of strategy *l* during the round trip time

*LT*^*h*^_*l*_ layover time of strategy *l* at the turning point or terminals

*N* set of stops of a single bus line

*N*_*l*_ set of stops related to strategy *l*

*q*_*l*,*p*_ vehicle’s capacity of strategy *l* departing from stop *p*

*RT*^*h*^_*l*_ total running time of strategy *l* in period *h*

*S* set of start stops of strategies

*t*_*b*_ average boarding time per passenger

*t*_*a*_ average alighting time per passenger

$V_{{\left(p,j\right)}_{p}^{+}}^{h}$ the number of passengers boarding at stop *p* and alighting at stop *j* in period *h*

$V_{{\left(i,p\right)}_{p}^{-}}^{h}$ the number of passengers boarding at stop *i* and alighting at stop *p* in period *h*

$V_{{\bar{\left(i,j\right)}}_{p}}^{h}$ the number of passengers passing through stop *p* who board at stop *i* and alight at stop *j* in period *h*

$WT_{p,j}^{\max}$ maximum acceptable waiting time of passengers from stop *p* to stop *j*

*δ*^*l*^_*p*_ binary decision variable; equals 1 if strategy *l* visits stop *p*, and otherwise 0

*θ* average time consumed by bus deceleration/acceleration as well as doors opening and closing time at each stop

### Operational strategies-based bus operation system

All start and end stops for applying operational strategies are assumed to be determined on the basis of passenger load profiles and strategy-related service characteristics. The short turn and a mixed strategies are initiated beyond the route’s departure terminal and or/terminated before its arrival terminal. Candidate short-turn points are usually the major route stops on the public transport network [2,3]; we assume that these points/stops allow for buses to turn around. We further have two assumptions related to passengers. First, that passengers arriving at a bus stop will board the first arriving bus if it is not full; otherwise will board the following bus [31–33]. Second, passengers would not transfer between vehicles operated by different strategies on the single bus line.

#### Bus round trip time

For a single bus line, the round trip time, *CT*^*h*^_*l*_, that it takes for a bus using strategy *l* to complete a full cycle of its route at operation period *h*, consists of running time, dwell time, and layover time:

$$CT_{l}^{h}=RT_{l}^{h}+DT_{l}^{h}+LT_{l}^{h}\;\forall h\in H,\forall l\in L$$ (1)

The running time of strategy *l* along the route at period *h*, *RT*^*h*^_*l*_, may vary between periods because of the dynamic, stochastic and uncertain nature of traffic. Generally speaking, the running time during peak hours is longer than during the off peak period. In this work, we divide the operational time window into several periods, and consider each hour as a single period. The layover time of strategy *l* during period *h*, *LT*^*h*^_*l*_, is determined according to the requirement of bus operation agencies.

In addition, *DT*^*h*^_*l*._, the total dwell time of strategy *l* along the route in period *h* that depends on passenger boarding and alighting times at each stop and average time *θ* consumed by bus deceleration/acceleration as well as doors opening and closing at each stop, is expressed as:

$$DT_{l}^{h}=\sum_{p\in N}\delta_{p}^{l}\left(DW_{l,p}^{h}+\theta \right)\;\forall h\in H,\forall l\in L$$ (2)

In Eq (2), *δ*^*l*^_*p*_ is a binary decision variable; it equals 1 if strategy *l* serves stop *p*, and 0 otherwise. The dwell time of strategy *l* at stop *p* can be saved if strategy *l* does not serve the stop *p*; that is, savings of passenger boarding and alighting times and the time associated with bus deceleration/acceleration and doors opening and closing at stop *p*.

The processes of boarding and alighting are considered simultaneously (different doors for boarding and alighting) and boarding and alighting flows are independent of each other. Therefore, the average passenger boarding and alighting times of strategy *l* at stop *p* and period *h*, *DW*^*h*^_*l*,*p*_, is the larger one between passenger boarding and alighting times at a stop (respectively denoted as *BT*^*h*^_*l*,*p*_ and *AT*^*h*^_*l*,*p*_) in period *h*, i.e.

$$DW_{l,p}^{h}=\frac{\max\left\{BT_{l,p}^{h},AT_{l,p}^{h}\right\}}{f_{l}^{}}\;\forall p\in N,\forall h\in H,\forall l\in L$$ (3)

where *BT*^*h*^_*l*,*p*_ and *AT*^*h*^_*l*,*p*_ can respectively be represented by (4), (5):

$$BT_{l,p}^{h}=\sum_{j\in N}V_{{\left(p,j\right)}_{p}^{+}}^{h}\frac{\delta_{p}^{l}\delta_{j}^{l}f_{l}^{}}{\sum_{k\in L}\left(\delta_{p}^{k}\delta_{j}^{k}f_{k}^{}\right)}t_{b}^{}\;\forall p\in N,\forall h\in H,\forall l\in L$$ (4)

$$AT_{l,p}^{h}=\sum_{i\in N}V_{{\left(i,p\right)}_{p}^{-}}^{h}\frac{\delta_{i}^{l}\delta_{p}^{l}f_{l}^{}}{\sum_{k\in L}\left(\delta_{i}^{k}\delta_{p}^{k}f_{k}^{}\right)}t_{a}^{}\;\forall p\in N,\forall h\in H,\forall l\in L$$ (5)

In Eqs (4) and (5) the number of passengers using each strategy is proportional to its relative frequency. The product term between the binary variables in Eqs (4) and (5) ensures that passenger demand between two stops can use a strategy only if this strategy is related to these two stops.

#### Optimization problem formulation

The use of operational strategies is considered for the reduction of the operation cost of a public transport system. The objective is to minimize the number of vehicles required for covering passenger demand while ensuring all passengers within an acceptable service level. The complete formulation of the optimization problem is as follows:

$$\underset{\left\{f_{l}^{},\delta_{p}^{l}\right\}}{Min}\sum_{l\in L}\left(RT_{l}^{h}+LT_{l}^{h}+\sum_{p\in N}\delta_{p}^{l}\left(\frac{\max\left\{\sum_{j\in N}V_{{\left(p,j\right)}_{p}^{+}}^{h}\frac{\delta_{p}^{l}\delta_{j}^{l}f_{l}^{}}{\sum_{k\in L}\left(\delta_{p}^{k}\delta_{j}^{k}f_{k}^{}\right)}t_{b}^{},\sum_{i\in N}V_{{\left(i,p\right)}_{p}^{-}}^{h}\frac{\delta_{i}^{l}\delta_{p}^{l}f_{l}^{}}{\sum_{k\in L}\left(\delta_{i}^{k}\delta_{p}^{k}f_{k}^{}\right)}t_{a}^{}\right\}}{f_{l}^{}}+\theta \right)\right)f_{l}^{}$$ (6)

Subject to

$$0\leq f_{l}^{}\;\forall l\in L$$ (7)

$$q_{l,p}^{}f_{l}^{}\geq \sum_{j\in N}V_{{\left(p,j\right)}_{p}^{+}}^{h}\frac{\delta_{p}^{l}\delta_{j}^{l}f_{l}^{}}{\sum_{k\in L}\left(\delta_{p}^{k}\delta_{j}^{k}f_{k}^{}\right)}+\sum_{i\in N}\sum_{j\in N}V_{{\bar{\left(i,j\right)}}_{p}^{}}^{h}\frac{\delta_{i}^{l}\delta_{j}^{l}f_{l}^{}}{\sum_{k\in L}\left(\delta_{i}^{k}\delta_{j}^{k}f_{k}^{}\right)}\;\forall l\in L,\forall p\in N$$ (8)

$$\frac{1}{\sum_{l\in L}\left(\delta_{p}^{l}\delta_{j}^{l}\right)f_{l}^{}}\leq WT_{p,j}^{\max}\;\forall p,j\in N$$ (9)

$$\delta_{p}^{l}\in \left\{0,1\right\}\;\forall p\in N,\forall l\in L$$ (10)

$$\delta_{p}^{l}=1\;if\;l=FRO,\;ST,\;\forall p\in N_{l}^{}$$ (11)

$$\delta_{p}^{l}=1\;if\;\forall p\in S\;\cup \;\forall p\in E,\;\forall l\in L$$ (12)

$$\delta_{p}^{l}+\delta_{p+1}^{l}+\delta_{p+2}^{l}\geq 1\;if\;l=LS,MLS\;\forall p\in \left\{1,\ldots ,n_{l}^{}-2\right\},$$ (13)

In this model, the objective function expression (6) is the sum of round trip times of feasible strategies multiplied by their respective frequencies *f*_*l*_. In the round trip time term, the first element is running time of a strategy; the second element is bus layover time at terminals or turning points; the third element is the total dwell times of a strategy at intermediate stops. Constraint (7) guarantees that frequencies of strategies are nonnegative. Constraint (8) makes sure the number of onboard passengers is less than the bus capacity. This constraint refers to onboard passengers at stop *p* when the bus departs from that stop. The first term on the right hand side of this constraint represents passengers boarding at stop *p*, and the second term is in-vehicle passengers passing through stop *p*. Constraint (9) maintains waiting time per passenger within an acceptable service level. Constraint (10) shows whether or not strategies serve stops; equals 1 if strategies serve stops, and otherwise 0. Constraint (11) ensures that all stops, including start and end stops of strategies and intermediate stops, can be visited by FRO and ST strategies. Constraint (12) makes sure that all strategies must visit their start and end stops. Constraint (13) guarantees that strategies involved with skipping stops do not skip more than three successive stops.

### Emissions estimation

The MOVES model is utilized in this study to calculate bus emission factors [27] before and after the use of operational strategies. Vehicle specific power (VSP) is used to define the emission factor for each type of driving behavior of MOVES, making it more accurate to quantify emission factors than using the speed or acceleration. The MOVES model is capable of estimating bus emissions (CO_2_, HC, CO, NO_x_, PM_2.5_, etc.) at multiple scales given different levels of input data. Numerous default parameters in the model become available, but only for the U.S. vehicle emissions estimation because of being collected in the U.S. region. Thus, users should modify default parameters and enter specific information of the local vehicle operation conditions for non-U.S. regions, especially in China [34]. Liu et al. [35] offered a detailed procedure for applying MOVES to estimate vehicle emission factors in Shanghai, China, by revising default parameters based on local vehicle operating characteristics; that is, from vehicle’s global positioning system (GPS), emission performance from China’s vehicle emission standards, and operating environment from the local statistical yearbook. These revised input parameters include operating mode distribution, emission inventory, vehicle age distribution, meteorology, types of fuels used, etc. In this study, we applied the same approach of Liu et al. [35], in revising MOVES, for calculating bus emission factors in Dalian, China.

Following the generation of bus emission factors by the revised MOVES model, the total emissions of each pollutant (CO_2_, HC, CO, NO_x_, PM_2.5_) are calculated in Eq (14).

$$E_{m}^{total}=\sum_{l\in L}EF_{l,m}LH_{l}f_{l}\;m\in \left\{CO_{2},\;HC,\;CO,\;NO_{x},\;PM_{2.5}\right\}$$ (14)

In Eq (14), the amount of pollutant *m* emissions for a bus operation system is estimated by summing pollutant *m* emissions, produced by all buses, using optimal operational strategies. The pollutant *m* emissions of buses using a strategy *l* are obtained by multiplying the emission factor of pollutant *m* (unit: g/km) *EF*_*l*_, by the average travel distance of a bus using strategy *l*, *LH*_*l*_, and by the frequency of strategy *l*, *f*_*l*_. The emission factor of pollutant *m* can be generated by the revised MOVES model as is described above in this subsection. *LH*_*l*_ and *f*_*l*_ are obtained by solving the proposed optimization model in Subsection 2.2.

## Case study

**Fig 2:**
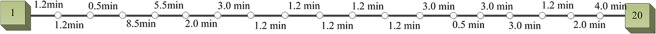
The topological structure of a real life transit route, Line 23 in Dalian.

The proposed methodology is performed on a real life bus line (Line 23) in Dalian, China. Line 23 runs from Dalian University of Technology (Stop 1) to Dalian University of Foreign Language (Stop 20), visiting 20 stops with a length of 13.8 km, as shown in Fig 2. It presents high demand due to its route along the main road connecting shopping centers (Heishijiao, Heping Square, Zhongshan Square), a tourist attraction (Xinhai Square), and the city center (Qinniwaqiao).

**Fig 3:**
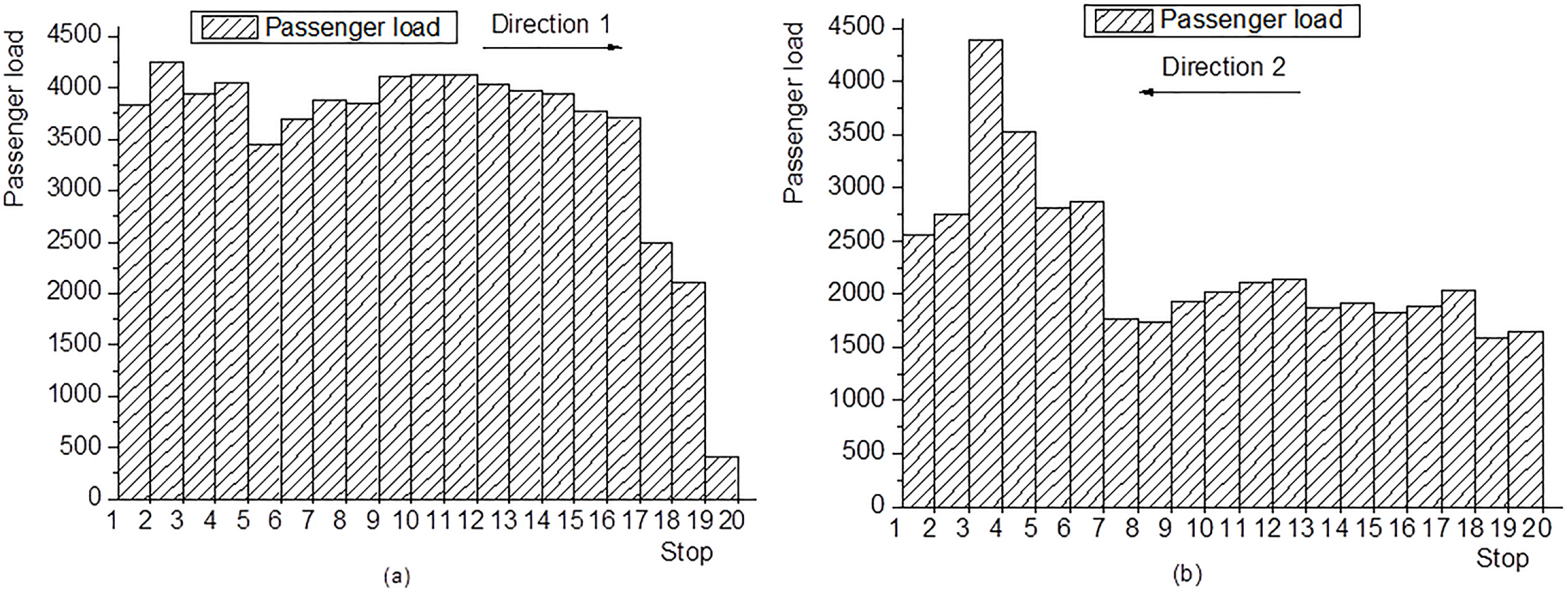
Passenger load profiles on bus Line 23. (a) Passenger load profile for Line 23 in Direction 1.(b) Passenger load profile for Line 23 in Direction 2.

Data was collected in the morning peak hour 7:00–8:00 on weekdays for an entire week of July 2015. Specifically, the collected demand data consists of alighting and boarding passengers that were manually counted at each stop for each bus trip. Average passenger boarding and alighting times were set at 2s and 1s per passenger, respectively. An average time is 0.3 min, consisting of bus deceleration/acceleration as well as door opening and closing times at each stop. The observed running times between two successive stops are shown in Fig 2. The layover time for each strategy is 2 min. The running time without serving any stop from stop 7 to stop 19 is 10 minutes for the short turn strategy. Based on the collected demand data, average load profiles are constructed, as shown in Fig 3.

As is illustrated by Fig 3 there is a high passenger demand between stop 1 and stop 19 for Line 23 in Direction 1, and between stop 1 and stop 7 in Direction 2. Additionally, max-load is observed on route segments 2–3 in Direction 1 and 4–3 in Direction 2; it is because stop 3, a transfer stop of Line 23, is located in a business office zone (Software Park Service Center). Based on the characteristics shown in Fig 3 it is possible to establish the sets of start and end stops of feasible operational strategies as is shown in Table 1.

**Table 1:** Start stops and end stops of feasible strategies.

Strategies	Direction 1		Direction 2	
	Start stop	End stop	Start stop	End stop
FRO	stop 1	stop 20	stop 20	stop 1
Limited stop	stop 1	stop 20	Stop 20	stop 1
Short turn	stop 1	stop 19	stop 7	stop 1
Mixed strategy	stop 1	stop 19	stop 7	stop 9

## Analysis and results

### Analysis of operational strategies-based bus system

**Fig 4:**
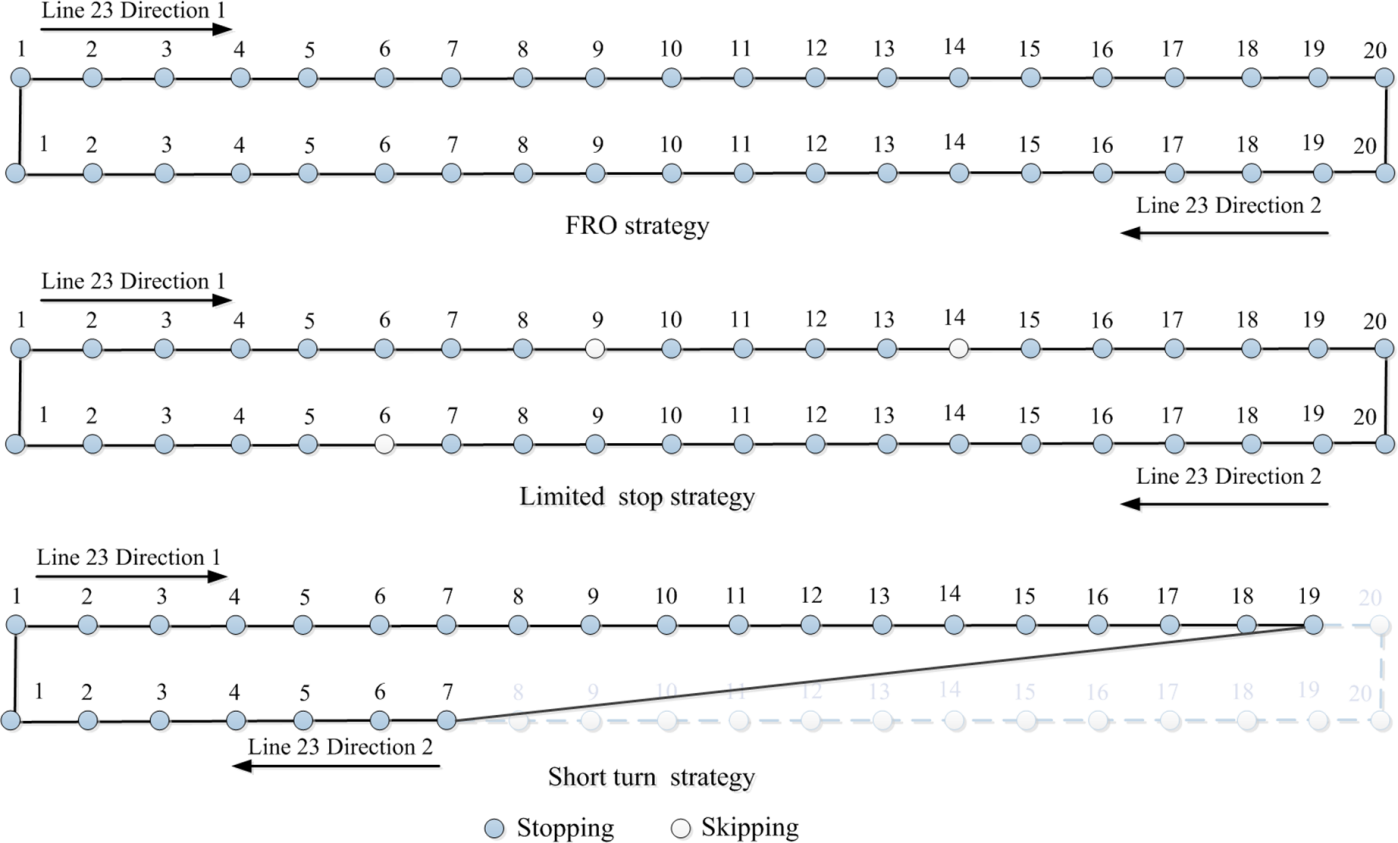
Topologies of optimal operational strategies.

The performance of the case study is evaluated by the use of vehicles required for covering all passenger demand. The operational strategies mathematical problem is formulated as a mixed integer non-linear programming (MINLP) problem. We used the outer approximation with both equality relaxation and augmented penalty (OA/ER/AP) algorithm coded in the DICOPT solver of GAMS to solve them [36,37]. Results of the system performance for both scenarios are shown in Table 2 and Fig 4.

**Table 2:** Bus system performance with and without operational strategies.

Scenarios	Type	Frequency (buses/h)	Round trip time (minutes)	Average speed (km/h)	fleet size (buses)
Operational strategies-based	FRO	18.11	119.13	13.90	91
	Short turn	33.20	83.51	14.46	
	Limited stop	4.20	117.51	14.09	
None	FRO	53.93	115.48	14.34	104

The optimal result of operational strategies-based bus system is to operate a combination of three strategies: FRO, short turn, and limited stop, with frequencies of 18.11, 33.20, and 4.20 buses/h, respectively. Fig 4 displays the service topological structures of these resulting operational strategies. As Table 2 results, the use of operational strategies improves significantly the bus system performance, and cuts the number of vehicles required by 12.5%.

Note some operational strategies such as the limited stop strategy in Table 2 and Fig 4 are not very practical, because they have low frequencies and present almost the same as FRO strategy. For a practical application of these operational strategies, the planner should take a closer look at the results to fix these situations. In this case, the limited stop strategy can be replaced by using the FRO strategy.

### Analysis of bus emissions

For the study, diesel buses, accounted for 60% in the Dalian bus system, have been selected. These diesel buses have been used around six years with a China IV emission standard. Bus operating conditions are extracted from a GPS installed in all vehicles. Urban unrestricted access is selected as the road type of Line 23. The Dalian meteorological bureau data for July 2015 is an average temperature of 25°C and humidity of 78%. Based on these local input data the MOVES model is modified for the Dalian region use. Five main pollutants are considered: CO_2_, HC, CO, NO_x_, PM_2.5_. Table 3 shows the emission factors of these five pollutants under two scenarios (with and w/o strategies) using the revised MOVES.

**Table 3:** Emission factors of two scenarios (g/km).

Scenarios	Type	Average speed (km/h)	CO_2_	HC	CO	NO_x_	PM_2.5_
Operational strategies-based	FRO	13.9	1740.78	0.26	0.75	5.60	0.37
	Short turn	14.46	1648.84	0.24	0.72	5.34	0.35
	Limited stop	14.09	1711.04	0.25	0.74	5.52	0.36
None	FRO	14.34	1668.63	0.25	0.73	5.40	0.35

Based on the estimated emission factors of pollutants in Table 3, the resulting frequencies using operational strategies and travel distance, the emissions of both scenarios in Table 4 are calculated using Eq (14). The results of operational strategies-based model are compared with the FRO strategy, without other strategies added, to illustrate reductions in emissions of approximately 13% for each of five pollutants: CO_2_, HC, CO, NO_x_, and PM_2.5_. The promising results indicate that the use of operational strategies can significantly reduce bus emissions and improve environment.

**Table 4:** Emissions (in kg) for the scenarios with and without the use of strategies.

Emissions	Without strategies	Using strategies	Reduction rate
CO_2_	2483.704	2169.846	12.64%
HC	0.365513	0.318898	12.75%
CO	1.080935	0.943191	12.74%
NO_x_	8.03235	7.007307	12.76%
PM_2.5_	0.52661	0.459763	12.69%

### Sensitivity analysis to parameter *θ*

When a bus skips a stop, it must result in a reduction of round trip time. This stems from the reduction in dwell time consisting of passenger boarding and alighting times as well as time denoted by parameter *θ*, generated by bus deceleration/acceleration as well as doors opening and closing at a stop. Variation in the value of parameter *θ* depends on bus performance and driving behavior, as well as traffic conditions. It is a factor that affects the use of operational strategies in a transit system.

**Fig 5:**
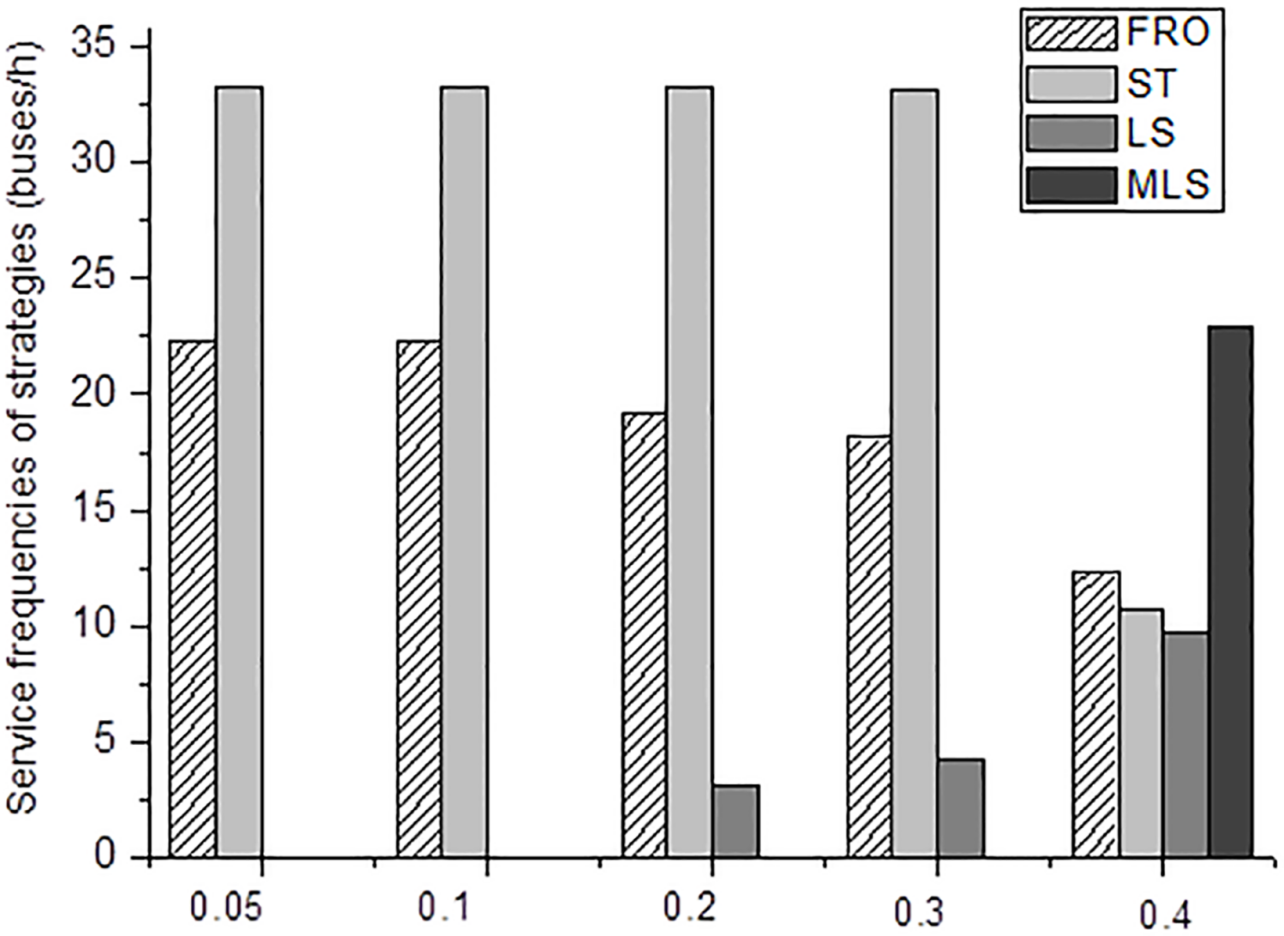
Results with different values of parameter *θ*.

In Fig 5, if the value of parameter *θ* is less than or equal to 0.1, the same strategy set, consisting of FRO and ST strategies, is obtained. When this value increases more than 0.2 and less than 0.4, LS strategy is added in the strategy set. This is because an increase in the value of parameter *θ* results in increasing round trip time. Using LS strategy can control the increase of round trip time by using its skipped stop option. In addition, the frequency of ST strategy almost remains the same, while the frequency of LS strategy presents an opposite trend to that of FRO strategy. As this value increases beyond 0.4, MLS strategy is introduced in the strategy set to further increase saving time in round trip time. It implies that for those routes with great time consumed by bus deceleration/acceleration as well as doors opening and closing at a stop, which may root in poor bus performance, bad driving behavior, or poor traffic conditions, strategies with serving a few stops presents more benefits in saving the number of vehicles required.

**Fig 6:**
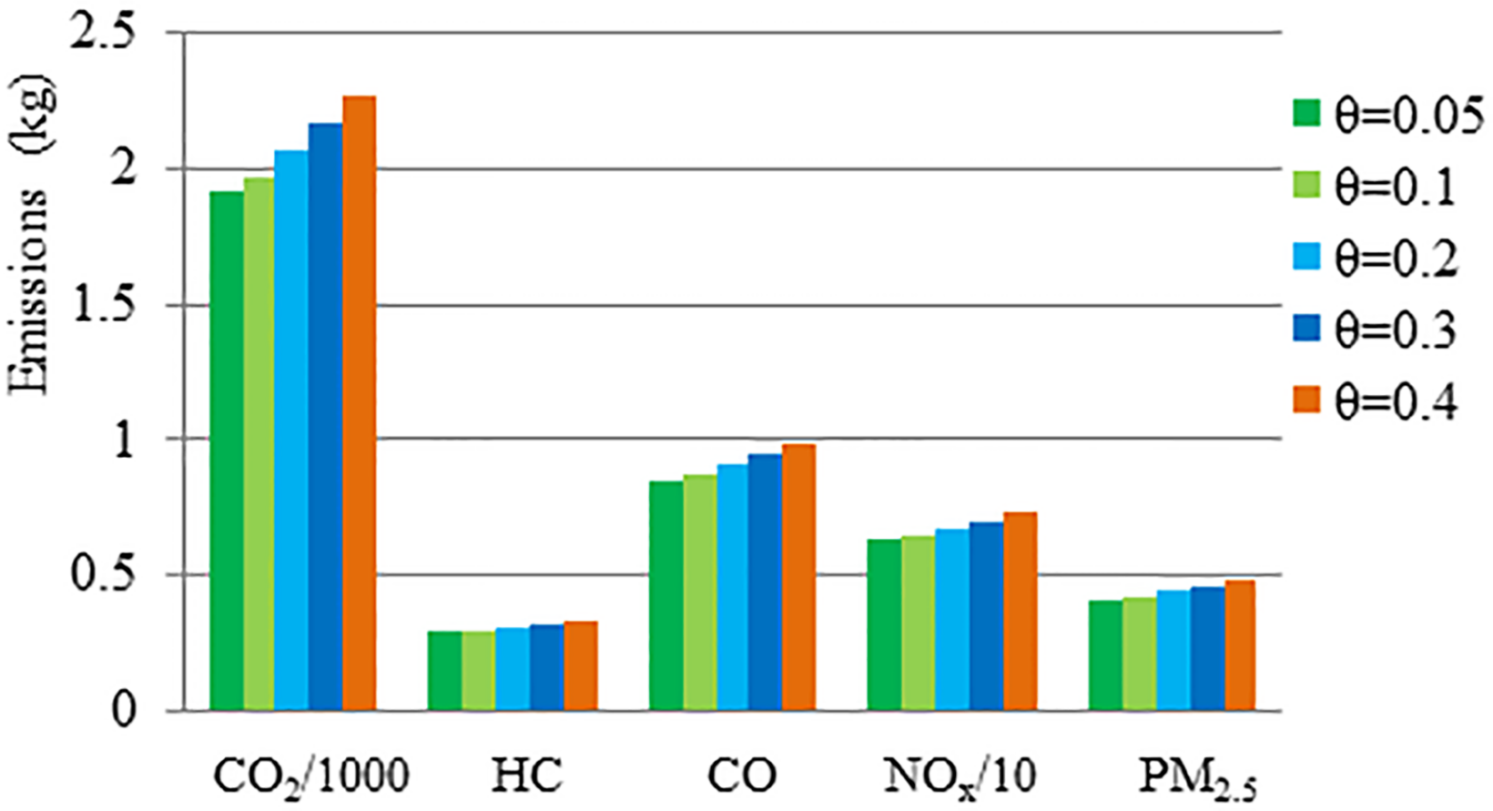
Emissions of strategies-based scenarios with different values of parameter *θ*.

Fig 6 illustrates the emissions for different values of the parameter *θ*. In this figure for *θ =* 0.05 the emissions of pollutants from the operational strategies-based model are the smallest. It rises with the increase *θ* from 0.05 to 0.4. This indicates that the reductions of emissions can be attained further by controlling bus performance related to deceleration/acceleration and doors opening and closing at stops; these are associated with a small value of *θ*.

Table 5 shows the percentage reduction for each pollutant using operational strategies in comparison with the FRO strategy (base case). The use of operational strategies reduces the emissions in an average sense by 12.37% across all scenarios with different *θ* values. It is apparent that for higher *θ* values this reduction increases. It implies that the use of strategies for poorly-performed buses can further improve the environment.

**Table 5:** Emission reduction in percentage by using operational strategies in comparison with the FRO strategy (base case), for different *θ* values.

Emissions	*θ*				
	0.05	0.1	0.2	0.3	0.4
CO_2_	11.64%	11.83%	12.20%	12.64%	13.00%
HC	11.89%	12.06%	12.37%	12.75%	13.06%
CO	11.87%	12.04%	12.36%	12.74%	13.05%
NO_x_	11.91%	12.07%	12.38%	12.76%	13.06%
PM_2.5_	11.76%	11.94%	12.28%	12.69%	13.03%

Note: emission reduction in percentage = (emissions without using strategies–emissions with strategies)/emissions without using strategies

### Effects of fare payment modes

In the real-life transit system, multiple fare payment modes are used. Table 6 shows the average estimated boarding and alighting times associated with the four different fare payment modes, i.e. on-board cash payment, contactless card, inserting coins, and off-board payment, which impacts bus dwell times and further influences bus operating cost as well as emissions. Nowadays, the contactless card has become more and more common. But it is not realistic to assume that every passenger has such a card. Above all, the tourists in a city usually do not have this type of cards for the local public transit services. Therefore, it is more often that we see a mixed system used in the real-life public transit system. For example, on a bus you may use either cash on board or a contactless card, which depends on your convenience. Besides, in some cities no changes will get back to you on the bus. In this case, the fare collection facility of this type of bus services is normally a toll collection machine and you shall put your money in it in the very front of the bus. This type of on-board cash payment (inserting coins) may incur much less time than the on-board cash mode with changes shown in Table 6 but shall be slightly higher than one corresponding to contactless card because passengers often do not get their money out of the pocket until they get on board. This paper does not consider the mixed system but focuses on the four payment modes listed in Table 6.

**Table 6:** Boarding and alighting times for each passenger under four payment modes.

Payment modes	Boarding time (s/person)	Alighting time (s/person)
On-board cash payment	10	1
Inserting coins	4.0	1
Contactless card	2.0	1
Off-board payment	1.5	1

**Fig 7:**
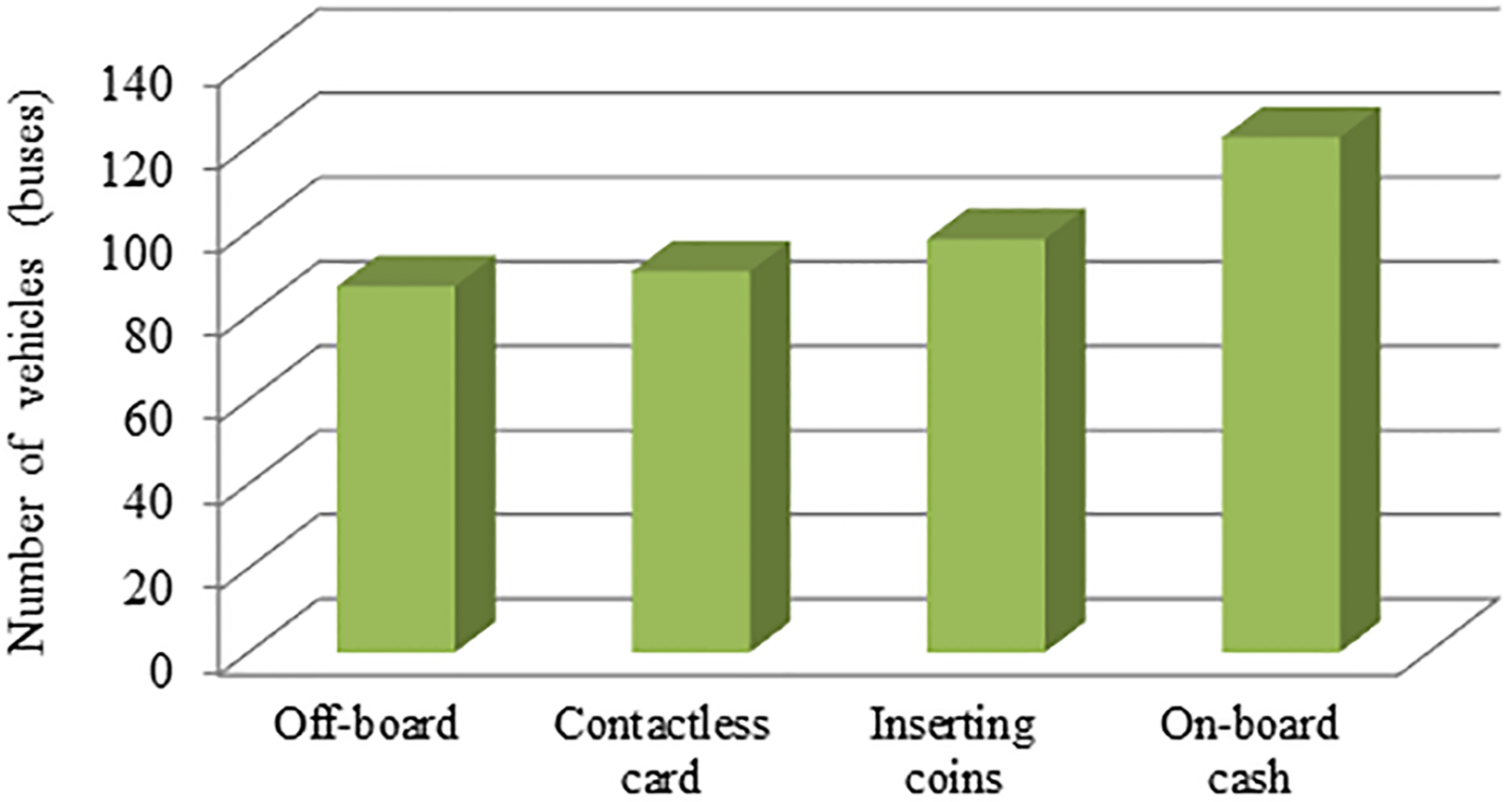
The number of vehicles for the strategies-based bus system under four payment modes.

Fig 7 represents the fleet sizes using the operational strategies-based bus system under four payment modes. Certainly the number of vehicles required for using the off-board payment mode is the minimum across all modes because of saving bus dwell and running times. Similar arguments hold for the other payment modes.

**Fig 8:**
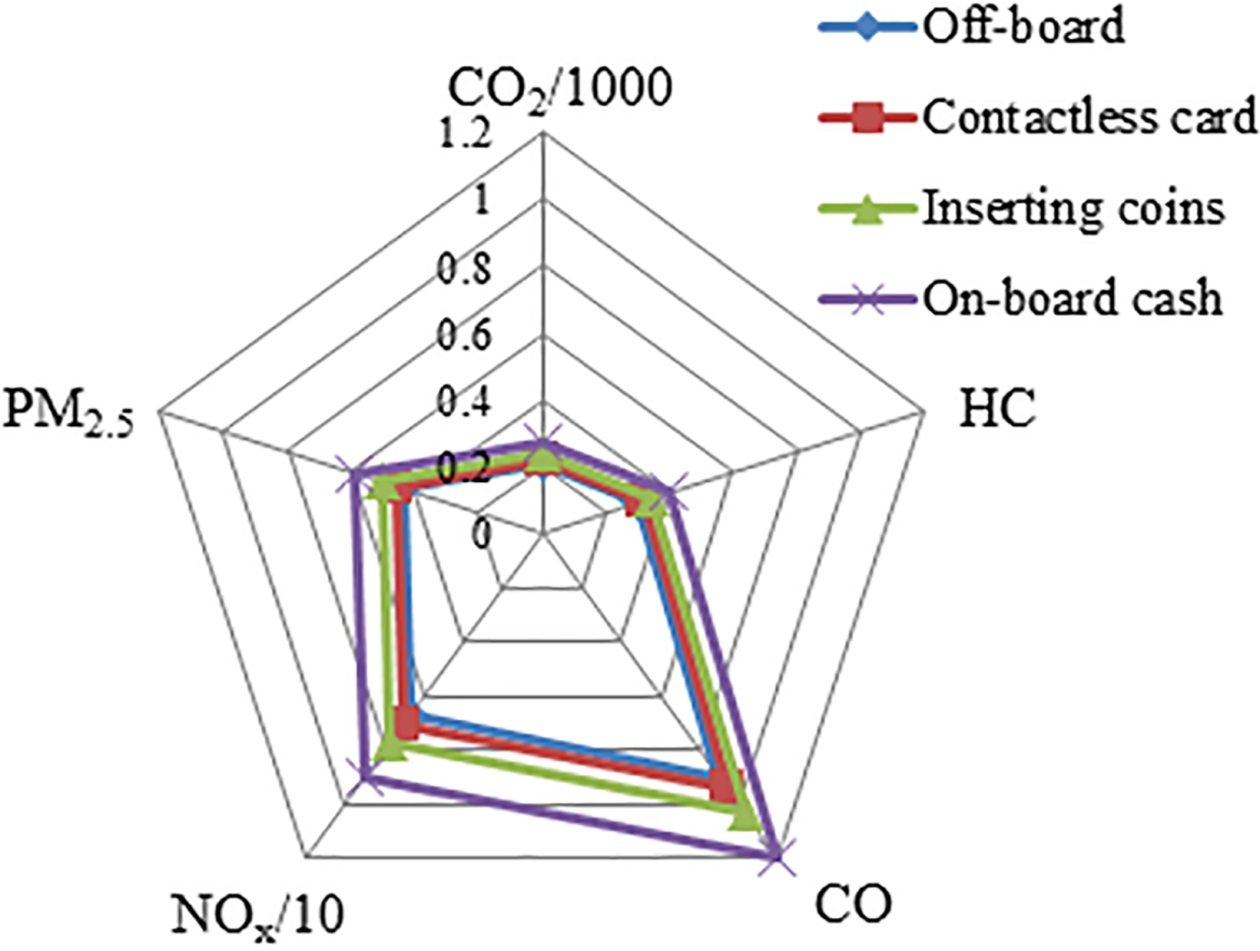
Emissions under four payment modes (kg).

Fig 8 shows the emissions of the bus system using different paying modes. It is observed that a cash payment mode is significantly higher, with emissions, than those using inserting coins, contactless card, or off-board payment mode, especially in pollutants NO_x_, PM_2.5_, and CO. The emissions for the transit system using the off-board payment mode are relatively close to that of contactless card mode. Table 7 contains the differences of emissions between using cash payment (base case) and other modes; for instance, the reduction percentage of emissions using inserting coins is more than 12%. A higher percentage reduction is observed for contactless card and off-board with more than 20%. That is, the use of contactless card payment will considerably reduce emissions.

**Table 7:** Emission reduction in percentage of payment modes compared with cash payment.

Payment modes	CO_2_	HC	CO	NO_x_	PM_2.5_
On-board cash payment	-	-	-	-	-
Inserting coins	12.21%	13.49%	13.89%	14.92%	13.85%
Contactless card	20.17%	20.82%	21.28%	22.22%	21.50%
Off-board payment	24.34%	24.53%	25.01%	25.84%	25.40%

The results of Fig 8 and Table 7 are of the case study in Dalian. It is to note that the number of passengers using contactless cards is not high because of only a few places to buy and recharge them; also, it offers only a discount of 5% where all bus routes have a flat fare of 1 or 2 RMB yuan. Thus, to save operating cost and improve the environment, fare makers should offer various fare-discount opportunities to encourage passengers to use contactless cards. Finally a point worth mentioning is that nowadays smartphones have become an essential part of our life including their payment technology. It is likely, therefore, that most of the passengers, including tourists, will use this mode of payment if it will be encouraged to use. This will not only be convenient, but will also improve the environment.

## Conclusion

The non-uniformity of public transport passenger demand warrants the use of operational strategies to attain operating efficiency. We proposed a methodology to analyze the benefits of using transit operational strategies to reduce operating cost and undesirable emissions. In this work, four alternative operational strategies are considered: full route operation (FRO), short turn (ST), limited stop (LS), and a mix of limited stop and short turn (MLS). A real life case study in Dalian of China is conducted to illustrate the application of this proposed methodology.

The reduction in the number of vehicles by 12.5% can be achieved when using operational strategies, in comparison with applying FRO strategy exclusively, which suggests operating efficiency improvement. The pollutant emission of transit vehicles are estimated by the MOVES emission model. We show that by applying operational strategies an approximately 13% reduction in emissions per pollutant (CO2, HC, CO, NOx, PM2.5) is attained, compared with the FRO scenario.

The value of parameter *θ* is the time associated with bus deceleration/acceleration and with doors opening and closing at a stop. Strategies serving a few stops, i.e., LS and MLS strategies, accommodate larger values of parameter *θ* because of poor bus performance, inappropriate driving behavior, or poor traffic conditions. The results of the case study show that the use of operational strategies reduces the emissions in an average sense by 12.37% across all scenarios with different *θ* values. It is apparent that for higher *θ* values this reduction increases. It implies that the use of strategies for poorly-performed buses can further improve the environment.

Moreover, this study contains the differences of emissions between using cash payment (base case) and other payment modes; for instance, in the case study the reduction percentage of emissions using inserting coins is more than 12%. A higher percentage reduction is observed for contactless card and off-board with more than 20%. That is, the use of contactless card payment will considerably reduce emissions.

Future research could extend the optimization model with user’s attributes consideration, such as values of travel time, ages, purposes of trip, and reliability measures required. The data of these attributes can be collected by smartphones using a big-data platform. The use of operational strategies can be also applied for improving electric bus operation’s efficiency. Nowadays the use of electric buses is growing rapidly to help the environment. However, there are issues around an efficient use of the limited battery capacity. Generally speaking, electric buses need to recharge during any daily operation resulting in a lower operation efficiency compared with the ordinary use of fuel buses. Therefore, it is timely to carry out research on these issues.

## Supporting information

S1 Appendix Demand for Line 23. (XLSX)

S2 Appendix MOVES Simulation for Line 23. (ZIP)
